# Sodium Metabisulfite Inhibits *Acanthamoeba* Trophozoite Growth through Thiamine Depletion

**DOI:** 10.3390/pathogens13060431

**Published:** 2024-05-21

**Authors:** Ronnie Mooney, Elisa Giammarini, Erin Corbett, Scott Thomson, Kevin McKinley, Paula Sinisterra Sebastian, Kiri Rodgers, Jana O’Donnell, Charles McGinness, Craig W. Roberts, Kanna Ramaesh, Fiona L. Henriquez

**Affiliations:** 1 School of Health and Life Sciences, University of the West of Scotland Lanarkshire, Glasgow G72 0LH, UK; ronnie.mooney@uws.ac.uk (R.M.);; 2 School of Computing, Engineering and Physical Sciences, University of the West of Scotland Paisley, Glasgow PA1 2BE, UK; 3 Strathclyde Institute of Pharmacy and Biomedical Sciences, University of Strathclyde, Glasgow G4 0RE, UK; 4 NHS Greater Glasgow and Clyde, Gartnavel Hospital, Department of Ophthalmology, Glasgow G12 0YN, UK

**Keywords:** *Acanthamoeba*, keratitis, amoeba, sodium metabisulfite, diagnosis, treatment, eye disease, protist, pathogen, thiamine

## Abstract

*Acanthamoeba* keratitis (AK) is a severe infection of the cornea. Prevention and treatment are difficult due to the inefficacy of currently available compounds. The impact of many commonly used compounds for routine examinations of *Acanthamoeba* is unexplored but might offer insight useful in combatting AK. In this study, we demonstrate that sodium metabisulfite, a common preservation constituent of eye care solutions, was found to be active against *Acanthamoeba* trophozoites at concentrations lower than that commonly found in eye drops (IC_50_ 0.03 mg/mL). We demonstrate that sodium metabisulfite depletes thiamine from growth medium and that *Acanthamoeba* is a thiamine auxotroph, requiring thiamine salvage for growth. The inhibitory effects of sodium metabisulfite can be overcome by thiamine supplementation. These results are consistent with the lack of key enzymes for thiamine biosynthesis in the genome of *Acanthamoeba*, an area which might prove exploitable using new or existing compounds. Indeed, this study highlights sodium metabisulfite as a useful inhibitor of *Acanthamoeba castellanii* trophozoites in vitro and that it acts, at least in part, by limiting available thiamine.

## 1. Introduction

*Acanthamoeba* keratitis (AK) is an infection that affects between 1 and 9 people per 100,000 contact lens wearers [1], with incidence rates increasing in several countries globally [2,3,4,5]. Patients typically present with severe eye pain, eye redness, blurred vision, sensitivity to light, and excessive tearing [6]. Clinical examination of the infected area necessitates the use of solutions containing mydriatic agents such as phenylephrine hydrochloride eye drops, and confirmation of AK is determined using culture-based, molecular, and microscopic analysis of patient lens cases or corneal scrapings [6,7]. The impact of routine compounds used in initial clinical examinations of *Acanthamoeba* spp. is as yet undescribed, but further investigation is warranted to inform diagnostic and therapeutic approaches and improve patient outcome.

Delayed diagnosis due to inconclusive results or failure of polymerase chain reaction (PCR) and/or culture to detect infection negatively impact the likelihood of resolving AK infections without surgical intervention [6,8]. Currently, treatment regimens for AK are arduous, typically consisting of prolonged combination therapies using polyhexamethylene biguanide (PHMB) or chlorhexidine and a diamidine such as propamidine or hexamidine [8]. Despite this, infection can be non-responsive to treatment, and corneal transplantation is often used to clear infection [9]. Identifying new preventative or therapeutic compounds to ease the burden of AK is important, and elucidating the role of existing compounds is useful in that it provides opportunity for compound repurposing or opens avenues for synergistic combinations to be trialled. Indeed, eye drops containing phenylephrine hydrochloride that are frequently used to dilate the pupil and facilitate fundus examination often contain excipients such as sodium metabisulfite [10,11,12,13,14,15], shown to have antimicrobial activity against organisms such as *Staphylococcus* spp., *Escherichia coli*, *Candida albicans*, and *Aspergillus flavus* [16,17] and widely used as a food preservative [18,19,20,21]. The antimicrobial mode of action of sodium metabisulfite is unclear, but it has been suggested that sulfite can react with various biological molecules. Interestingly, sodium metabisulfite has been shown to degrade thiamine in solutions [22], potentially limiting thiamine availability, resulting in an indirect antimicrobial effect. Thiamine is an essential micronutrient and serves as a co-factor for many enzymatic reactions involved in energy production and the synthesis of downstream metabolites [23]. Here, we investigate the antimicrobial activity of these compounds against *Acanthamoeba* at concentrations comparable to those found in commonly used eye drop solutions (e.g., phenylephrine hydrochloride; 1 mg/mL sodium metabisulfite).

This research demonstrates that sodium metabisulfite, a constituent of eye care products, can restrict the growth of *A. castellanii*. Through culture growth assays, bioinformatic analysis, and LC-MS analysis we demonstrate that *Acanthamoeba* is a thiamine auxotroph, scavenging this nutrient from the environment. The data presented here provide a platform from which potential new antimicrobial therapies and preventatives can be developed and incorporated into synergistic combinations that aid in the prevention and treatment of those suffering with AK. Furthermore, the inhibitory effect of sodium metabisulfite has the potential to influence and delay accurate diagnosis of the disease, ultimately affecting disease outcome, and so should be considered during initial screening.

## 2. Materials and Methods

### 2.1. Acanthamoeba Species

*Acanthamoeba castellanii* (ATCC 50370) trophozoites were maintained in Proteose Peptone and Glucose (PG)-supplemented medium [24]. Prior to experimental work, trophozoites were transferred to the chemically defined medium M20 [25] containing a thiamine concentration of either 1.25 mg/mL [25] or 0.0078 mg/mL (minimum concentration to support growth) and incubated at 25 °C for four days to allow cell acclimatisation.

### 2.2. Determination of Minimum Thiamine Concentration for A. castellanii Growth

*A. castellanii* trophozoites (1 × 10^4^ cells/mL) were seeded in chemically defined medium M20 [25] lacking thiamine or with thiamine concentrations of 1.25 mg/mL, 0.75 mg/mL, 0.0078 mg/mL, or 0.00093 mg/mL, and incubated at 25 °C. Growth was monitored by enumerating *A. castellanii* trophozoites using a haemocytometer (BlauBrand—Brand GMBH, Wertheim, Germany) and the data were recorded daily in a 24 h interval for 4 days (96 h in total).

### 2.3. Sodium Metabisulfite Acanthamoeba Inhibition Assay

*A. castellanii* trophozoites (1 × 10^5^ cells/mL) were seeded in standard M20 (with 1.250 mg/mL thiamine) [25] or modified M20 (with 0.0078 mg/mL thiamine). Sodium metabisulfite (40 mg/mL) (Sigma, Poole, UK) was added at a starting concentration of 20 mg/mL and serially diluted to a final concentration of 0.156 mg/mL and incubated for 96 h at 25 °C. *Acanthamoeba* viability was assessed by Alamar Blue™ (Thermo Fisher Scientific, Renfrew, UK) after the incubation time for a further 24 h [26].

### 2.4. Thiamine Chromatography

Sodium metabisulfite (Sigma Aldrich, Dorset, UK) was added to PG and M20 media in concentrations of 10 mg/mL, 2.5 mg/mL, 1.25 mg/mL, and 0.156 mg/mL and to the 10 ppm thiamine standard for a final sodium metabisulfite concentration of 10 mg/mL. Thiamine, thiamine/sodium metabisulfite, media only, and media/sodium metabisulfite solutions were incubated at room temperature for 24 h in the absence of light. An Agilent 1260 Infinity HPLC coupled to an Agilent 6120 Quadrupole MS was used for all analyses. Agilent OpenLab software v2.8 was used for instrument control and data analysis. Separation of analytes was performed on a Gemini 5u C18 110A column (150 × 4.6 mm, 5 µm particle size) (Phenomenex, Macclesfield, UK). Column temperature was set to 30 °C. The mobile phase was 20 mM ammonium acetate and 0.1% *v*/*v* forming acid in water at a flow rate of 1 mL/min, and sample injection volume was 20 µL. Total run time of the method was 8 min. The ion source was atmospheric pressure ionisation—electrospray (API-ES) in positive mode. Spray chamber conditions were as follows: drying gas flow—12 L/min; nebuliser pressure—35 psig; drying gas temperature—250 °C; capillary voltage—3000 V. Experiments were performed in single-ion monitoring (SIM) mode, with a target SIM ion *m*/*z* 265.1 and dwell time of 290 ms. 

### 2.5. In Silico Analysis

The protein database (www.ncbi.nlm.nih.gov, accessed on 16 February, 2024) and the genome database for *A. castellanii* (www.amoebadb.org, accessed on 16 February, 2024) were mined for proteins involved in thiamine biosynthesis. Names of characterised proteins involved in thiamine biosynthesis from other kingdoms were used as queries for BLAST searches. Hits with low expectation values (<0.01) were subjected to further back-BLAST to assign putative functions by mining the NCBI protein database for sequences closely aligned to those available on Amoebadb. If back-BLAST results were in agreement with the queried protein but had an E-value below 0.01 and/or a percentage identical score below 40%, sequences were aligned using the Muscle alignment tool [27] and visualised using Jalview v2.11.3.2 [28,29]. Upon alignment, sequences were screened for the presence of active sites, and proteins lacking key active sites were considered false.

### 2.6. Statistical Analysis

Statistical significance between concentrations was determined by using a two-sided, two-sample t-test with a type I error rate of 0.05 in Microsoft Excel. ANOVA single-factor analysis, with alpha = 0.01, was conducted in Microsoft Excel, based on the cell viability values obtained. For all work undertaken in this study, a total of 3 independent experiments were conducted, with each independent experiment performed in triplicate.

## 3. Results

### 3.1. Sodium Metabisulfite Inhibits the Growth of A. castellanii Trophozoites

**Figure 1:**
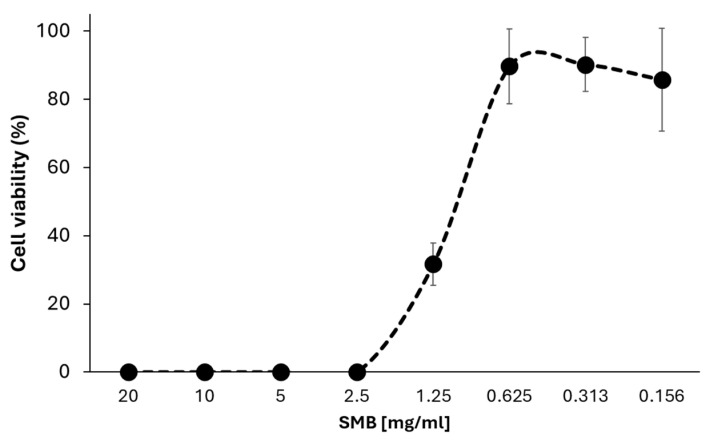
*A. castellanii* trophozoites (1 × 10^4^ cells/mL) in each well were seeded in M20 with a thiamine concentration of 1.25 mg/mL (closed circle) and sodium metabisulfite doubly diluted from 20 mg/mL to 0.156 mg/mL for 96 h. Data are mean cell count ± SD; n = 3 independent experiments performed in triplicates.

Sodium metabisulfite is a constituent of several eye care solutions [11,12,13,14,15] and has been shown to have antimicrobial properties in other organisms [16,17]. Further investigation examined the effect of different concentrations of this compound on *Acanthamoeba* growth (Figure 1). Sodium metabisulfite was found to inhibit the growth of *Acanthamoeba* with an IC_50_ of 1.21 mg/mL (Figure 1).

### 3.2. Acanthamoeba Trophozoites Require Thiamine for Growth

**Figure 2:**
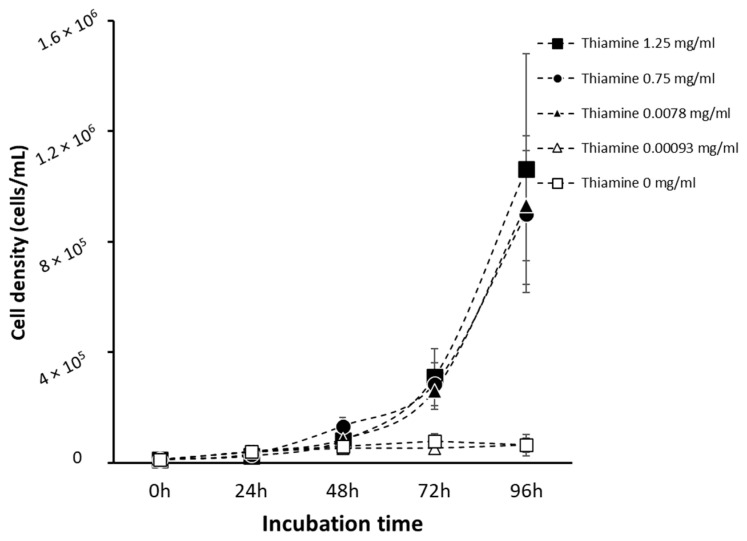
Growth curve of *A. castellanii* (1 × 10^4^ cells/mL) in chemically defined medium M20 containing variable levels of thiamine. Thiamine concentrations ranged from 1.25 mg/mL to 0 mg/mL. Cell density was estimated over 96 h in cells incubated with 1.25 mg/mL (closed square), 0.75 mg/mL (closed circle), 0.0078 mg/mL (closed triangle), 0.00093 mg/mL (open triangle), and 0 mg/mL (open square) of thiamine. Data are mean cell count ± SD; n = 3 independent experiments performed in triplicates.

Sodium metabisulfite has been shown to degrade thiamine in infusion solutions [22]. To assess the role of thiamine depletion on *A. castellanii*, it is necessary to define the minimum concentration of thiamine required to support *Acanthamoeba* growth. Trophozoites (1 × 10^4^ cells/mL), seeded in M20 medium with varying concentrations of thiamine (Figure 2), were monitored daily for 96 h. Cell counts show a significant increase in the growth of *A. castellanii* at thiamine concentrations above 0.0078 mg/mL (*p* < 0.05). Cell growth was significantly inhibited at lower concentrations of thiamine (*p* < 0.05), suggesting a reliance on exogenous thiamine for cell growth (Figure 2). Growth medium containing 0.0078 mg/mL of thiamine was established as a minimal medium capable of sustaining *Acanthamoeba* growth.

### 3.3. Sodium Metabisulfite Inhibits Acanthamoeba Growth through Degradation of Thiamine

**Figure 3:**
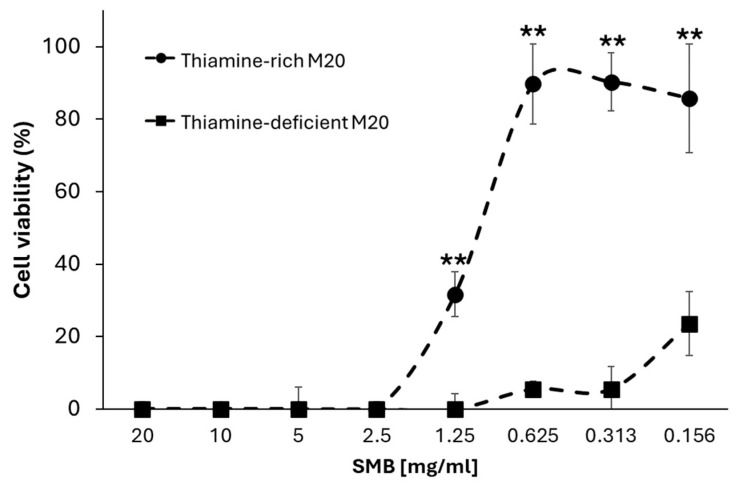
*A. castellanii* trophozoites (1 × 10^4^ cells/mL) in each well were seeded in M20 with a thiamine concentration of 1.25 mg/mL (closed circle) and 0.0078 mg/mL (closed square), respectively, and sodium metabisulfite doubly diluted from 20 mg/mL to 0.156 mg/mL for 96 h. Efficacy of sodium metabisulfite was significantly improved in thiamine-deficient medium (*p* < 0.01) relative to cells incubated in thiamine-rich medium. Student’s *t*-test, *p* < 0.01 is indicated by “**”; data are mean viability ± SD; n = 3 independent experiments performed in triplicates.

**Figure 4:**
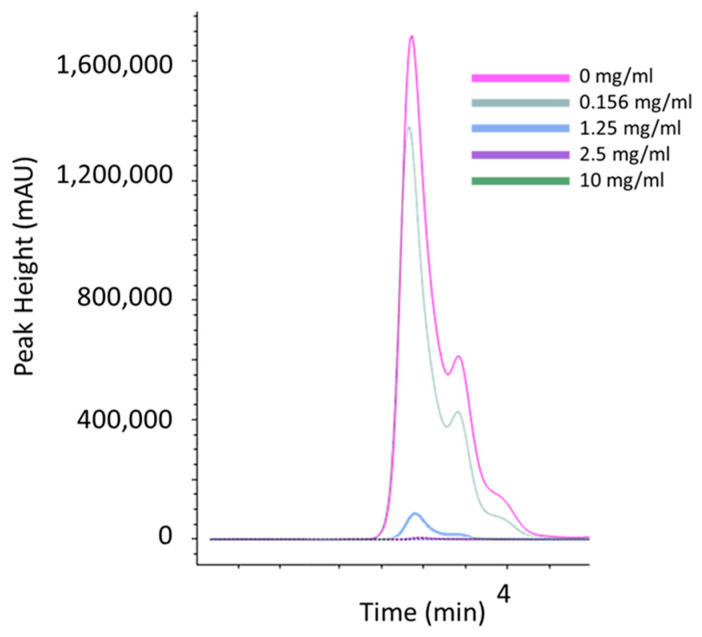
LC-MS was used to detect thiamine levels in thiamine-rich M20 medium (1.25 mg/mL) after 24 h incubation with or without sodium metabisulfite at concentrations of 0 mg/mL (pink line), 0.156 mg/mL (grey line), 1.25 mg/mL (blue line), 2.5 mg/mL (purple line), and 10 mg/mL (green line). Peak height is indicative of available thiamine in the medium. Thiamine is depleted in M20 medium in a dose-dependent manner when incubated with sodium metabisulfite.

*A. castellanii* trophozoites were seeded in either thiamine-rich (thiamine exceeding growth requirements) M20 medium (1.25 mg/mL) or thiamine-minimal M20 medium (0.0078 mg/mL) for 96 h with doubly diluted concentrations of sodium metabisulfite (from 20 mg/mL to 0.156 mg/mL). Sodium metabisulfite inhibited *A. castellanii* trophozoites in a dose-dependent manner in both medium types (Figure 3). Amoebae incubated in thiamine-rich M20 showed a significantly higher tolerance to sodium metabisulfite (40.3-fold higher, *p* < 0.01) than those in thiamine-minimal M20 (Figure 3; IC_50_: 1.21 mg/mL and 0.03 mg/mL, respectively), suggesting that thiamine depletion is in part responsible for sodium metabisulfite efficacy against *A castellanii*. Supporting this observation, the thiamine levels in thiamine-rich M20 medium (Figure 4 and Figure S2a), PG medium (Figure S2b), and a thiamine standard (Figure S2c) were depleted in the presence of sodium metabisulfite in a dose-dependent manner.

### 3.4. Acanthamoeba Is a Thiamine Auxotroph

**Figure 5:**
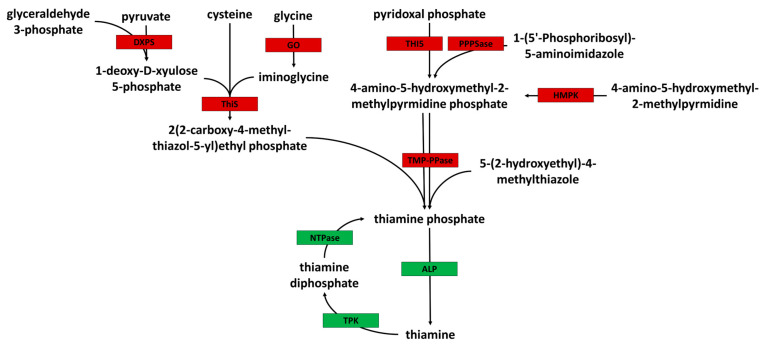
Key enzymes involved in the de novo synthesis of thiamine were identified and their presence (green box) or absence (red box) within the genome of *A. castellanii* predicted using BLAST. In silico analysis revealed that *A. castellanii* lacked several upstream enzymes necessary for the synthesis of thiamine from precursory metabolites and that it likely relies on thiamine salvage. Enzyme key: DXPS—1-deoxy-D-xylulose-5-phosphate synthase, GO—glycine oxidase, ThiS—thiazole synthase, THI5—pyrimidine precursor biosynthesis enzyme, PPPSase—phosphomethylpyrimidine synthase, HMPK—hydroxymethylpyrimidine kinase, TMP-PPase—thiaminephosphate pyrophosphorylase, ALP—alkaline phosphatase, TPK—thiamine pyrophosphokinase, NTPase—Nucleoside-triphosphatase.

Detailed in silico analysis of the *A. castellanii* genome was carried out to identify enzymes necessary for the de novo synthesis of thiamine (Table S1). The data suggest that *Acanthamoeba* is unable to synthesize thiamine de novo. In brief, BLAST analysis suggests that *Acanthamoeba* does not possess a thiamine phosphate pyrophosphorylase enzyme necessary for the synthesis of thiamine phosphate from 4-amino-5-hydroxymethyl-2-methylpyrmidine diphosphate together with either 2(2-carboxy-4-methyl-thiazol-5-yl)ethyl phosphate or 5-(2-hydroxyethyl)-methylthiazole. However, alkaline phosphatase enzymes were identified (ACA1_194910 and ACA1_182290) for the conversion of thiamine phosphate to thiamine (Figure 5), as were thiamine pyrophosphokinase enzymes necessary for the conversion of thiamine to the active thiamine diphosphate (ACA1_225950 and ACA1_050760). Despite this, genome mining revealed no evidence of the cysteine-dependent adenosine diphosphate thiazole synthase enzyme required for the synthesis of 2(2-carboxy-4-methyl-thiazol-5-yl)ethyl phosphate from glycine and the thiazole synthase and thiazole tautomerase enzymes necessary for the synthesis of 2(2-carboxy-4-methyl-thiazol-5-yl)ethyl phosphate from iminoglycine, 1-deoxy-D-xyulose 5-phosphate, and cysteine. More significantly, we found no evidence of a hydroxymethylpyrimidine kinase gene in the *A. castellanii* genome, essential in the synthesis of the 4-amino-5-hydroxymethyl-2-methylpyrmidine diphosphate metabolite from 4-amino-5-hydroxymethyl-2-methylpyrmidine phosphate (Figure 5), with the putatively annotated gene ACA1_061800 lacking almost all functional sites described in the literature (Figure S1) [30].

## 4. Discussion

Prevention and treatment of AK is difficult due to the relatively limited understanding of the infectious organisms’ biochemical requirements. Thus, expanding this knowledge can aid in the development of a more targeted approach. The impact of many commercially available compounds on *Acanthamoeba* is yet to be explored, yet understanding these interactions provides a potential route for compound repurposing using solutions already deemed safe for use in humans [31]. Compounds commonly used for mydriasis often contain sodium metabisulfite [11,12,13,14,15] and have been shown to possess antimicrobial activity in other organisms [16,17]. Here, we have provided evidence to demonstrate that this compound can also reduce *Acanthamoeba* trophozoite viability after 96 h.

Sodium metabisulfite, a salt of sulphurous acid recognised by the European Commission under regulation 1129/2011, is extensively used for the preservation of fresh and dried fruit, vegetables, juices, and wines due to its antimicrobial properties [18,19,20,21]. Sodium metabisulfite has previously been shown to have antifungal (e.g., *Aspergillus* spp., *Fusarium* spp., *Rhizoctonia solani*) and antibacterial (e.g., *Staphylococcus* spp., *Escherichia coli*, *Pseudomonas aeruginosa*, *Bacillus* spp.) capabilities [32,33,34,35,36]. The broad-spectrum antimicrobial activity of sodium metabisulfite and its use in eye care solutions warranted further investigation into its activity against *Acanthamoeba* species. To the best of our knowledge, this is the first study investigating the antimicrobial activity and potential mode of action of sodium metabisulfite against any *Acanthamoeba* species. The activity of sodium metabisulfite against *A. castellanii* trophozoites was initially investigated using standard M20 medium [25], containing 1.25 mg/mL of thiamine, and a concentration of 1.21 mg/mL of sodium metabisulfite was necessary to reduce cell viability by 50%. To further elucidate the mechanisms by which sodium metabisulfite acted on *Acanthamoeba,* we built on work previously undertaken by Scheiner and colleagues [22], in which sodium metabisulfite was shown to reduce the availability of thiamine in infusion solutions. Thiamine is an essential micronutrient and is converted to the active thiamine diphosphate via the activity of thiamine pyrophosphokinase, at which point it can act as a co-factor for enzymes necessary for energy production and normal cellular function [37]. For example, the tricarboxylic acid (TCA) cycle-associated enzymes pyruvate dehydrogenase and 2-oxoglutarate dehydrogenase, necessary for the conversion of pyruvate to acetyl-CoA and pyruvate dehydrogenase to succinyl-CoA, respectively, are reliant on thiamine diphosphate as a co-factor [37,38,39]. Similarly, fatty acid degradation and the pentose phosphate pathway also rely on this co-factor for the synthesis of essential metabolites or as a means of producing energy through α-oxidation and β-oxidation, or glycolysis [37,40,41]. We noted that the depletion of available thiamine in growth media resulted in a decrease in cell growth, with no growth observed in M20 medium containing less than 0.0078 mg/mL of thiamine, suggesting that *Acanthamoeba* was highly reliant on thiamine supplementation and perhaps lacked one or more necessary proteins for the de novo synthesis of the compound. Indeed, while our in silico analysis of the *Acanthamoeba* genome did suggest that it possesses thiamine pyrophosphokinase enzymes for the synthesis of thiamine diphosphate, we found no evidence of protein sequences corresponding to essential upstream enzymes, for example, hydroxymethylpyrimidine kinase or thiamine phosphate pyrophosphorylase, necessary for de novo synthesis of thiamine [42], which supported our finding that the scavenging of thiamine from the environment is essential for cell function.

In agreement with Scheiner et al. [22], we found that supplementing M20 medium with sodium metabisulfite resulted in a dose-dependent decrease in thiamine. To demonstrate that this interaction was influencing cell viability, the same cytotoxicity assay was performed on cells incubated in M20 medium containing the minimal thiamine concentration necessary for survival (0.0078 mg/mL). A 40-fold decrease in the IC_50_ was noted relative to the thiamine-rich medium used initially (0.03 mg/mL and 1.21 mg/mL, respectively). The results of this study suggest that sodium metabisulfite acts on *Acanthamoeba* through the depletion of available thiamine. It is interesting to note that the concentrations of sodium metabisulfite capable of inhibiting *A. castellanii* growth were comparable to those listed in commonly used eye care solutions (1 mg/mL), suggesting it may have use in the prevention of AK, perhaps through integration into contact lens solutions, particularly given the lack of toxicity in ocular studies [31]. Conversely, it could be speculated that the activity of the compound on *Acanthamoeba* may influence diagnosis, with mydriatic agents containing sodium metabisulfite used for initial diagnostics perhaps reducing the culturability of isolated samples. In both instances, significantly more research is required.

In summary, this study highlights that excipients in eye care solutions such as sodium metabisulfite can have inhibitory effects on *Acanthamoeba* trophozoites that could be exploited in preventative or therapeutic strategies against AK. It should also be considered that use of these compounds during initial diagnostics could interfere with the accurate detection of *Acanthamoeba*. Further understanding of how these constituents impact amoeba growth and how these compounds act collectively is important, and the synergy of compounds such as sodium metabisulfite with other widely used compounds should be assessed. Overall, we have demonstrated that sodium metabisulfite is an effective inhibitor of *A. castellanii* growth and that the organism lacks the molecular tools necessary for de novo thiamine synthesis, presenting a potential area for future drug targeting.

## Data Availability

Data are contained within the article.

## Supplementary Materials

The following supporting information can be downloaded at: https://www.mdpi.com/article/10.3390/pathogens13060431/s1, Table S1: BLAST queries and accession numbers; Figure S1: Sequence alignment for HMPK, Figure S2: (a) Thiamine depletion in M20 medium in the presence of sodium metabisulfite; (b) Thiamine depletion in PG medium in the presence of sodium metabisulfite; (c) Depletion of a thiamine standard in the presence of sodium metabisulfite.
