# Supplementary material for: Sodium Metabisulfite Inhibits Acanthamoeba Trophozoite Growth through Thiamine Depletion
Source: Pathogens. 2024 May 21;13(6):431. doi: 10.3390/pathogens13060431 (PMC11206890; doi:10.3390/pathogens13060431)
Supplement: Supplementary file 1 [file pathogens-13-00431-s001.zip › Supplementary figures Word.pdf]

Table S1: BLAST queries and accession numbers.

Query	Sub-query	BLAST hits (E-value <0.01)	Amoebadb ID	AmoebaDB annotation	Back-BLAST (Most common hits)	Accession number	E-value <0.01 and Per. ID > 40%
1-deoxy-D-xylulose-5-phosphate synthase	Pseudomonas fluorescens SBW25 CAI2799601.1	2	ACA1_095900	Hypothetical protein	Pyruvate dehydrogenase	XP_004334943.1	Yes
			ACA1_292910	branched-chain alpha-KETO ACID decarboxylase E1 beta subunit, putative	Hypothetical protein/2-oxoisovalerate dehydrogenase	XP_004368155.1	Yes
	1-deoxy-D-xylulose-5-phosphate synthase, chloroplastic [Medicago truncatula] NP_001408947.1	3	ACA1_292910				
			ACA1_095900				
			ACA1_287820	Transketolase	Transketolase	XP_004367836.1	Yes
	1-deoxy-D-xylulose 5-phosphate synthase [Syncephalis pseudoplumigaleata] RKP28295.1	3	ACA1_292910				
			ACA1_095900				
			ACA1_287820				
Glycine oxidase	glycine oxidase [Pseudanabaena biceps PCC 7429] ELS30590.1	5	ACA1_153640	Dehydrogenase	FAD-Dependent oxidoreductase	XP_004353662.1	Yes
			ACA1_1.3420	L-2-hydroxyglutarate dehydrogenase, mitochondrial precursor, putative	Hypothetical protein/FAD-dependent oxidoreductase	XP_004345952.1	Yes

			ACA1_253480	FAD dependent oxidoreductase domain containing protein	FAD-Dependent oxidoreductase	XP_004367617.1	Yes
			ACA1_052070	.	FAD-Dependent oxidoreductase	XP_004336967.1	Yes
			ACA1_012850	FAD dependent oxidoreductase	FAD-Dependent oxidoreductase	XP_004342374.1	Yes
	2-Hydroxyacid oxidase 2 [Mus musculus] NP_062418.3	0					
	Glycine oxidase [Lachnellula suecica] TVY85280.1	2	ACA1_253480				
			ACA1_133460	MACRO domain containing 2 isoform 1, putative	ADP-ribose glycohydrolase	XP_004341933.1	Yes
Thiazole synthase	Thiazole synthase [Saccharomyces boulardii (nom. inval.)] KQC43750.1	3	ACA1_327700	hypothetical protein	Monoamine oxidase	XP_004333109.1	Yes
			ACA1_352610	Hypothetical protein	Hypothetical protein	XP_004342818.1	No
			ACA1_097590	amine oxidase, flavincontaining superfamily protein	Amine oxidase	XP_004335086.1	No
	Thiazole Synthase [Bacillus sp. BR3(2024)] WP_342761659.1	1	ACA1_232380	thiazole biosynthesis protein ThiG, putative	Pyridoxal 5'-phosphate synthase lyase	XP_004340966.1	Yes
	Thiazole synthase [Labeo rohita] KAI2645569.1	0					
Pyrimidine precursor biosynthesis enzyme	Pyrimidine precursor biosynthesis enzyme	0					

	[Metschnikowia aff. pulcherrima] QBM87846.1						
	Pyrimidine precursor biosynthesis enzyme [Geobacillus thermodenitrificans] ARP43168.1	0					
	Pyrimidine precursor biosynthesis enzyme THI5 [Striga asiatica] GER28095.1	0					
Hydroxymethyl pyrmidine kinase	Hydroxymethylpyrimidine kinase; phosphomethylpyrimidine kinase [Intrasporangium calvum DSM 43043] ADU47915.1	1	ACA1_061800	phosphomethylpyrimidine kinase	Bifunctional hydroxymethylpyrimidine kinase/phosphomethylpyrimidine kinase	XP_004339452.1	No
	Hydroxymethylpyrimidine/phosphomethylpyrimidine kinase-like [Ylistrum balloti] >XP_060084823.1	2	ACA1_061800				
			ACA1_376300	pyridoxal kinase	Pyridoxal kinase	XP_004353697.1	Yes
	Hydroxymethylpyrimidine kinase, partial [Musa troglodytarum] URE09993.1	3	ACA1_061790	thiaminephosphate pyrophosphorylase	Hydroxyethylthiazole kinase	XP_004339451.1	Yes
			ACA1_061800				
			ACA1_376300				
Thiamine phosphate pyrophosphorylase	Thiamine phosphate pyrophosphorylase [Salmonella enterica subsp. enterica serovar Typhimurium str. LT2] NP_463032.1	1	ACA1_061790				

	Thiamine-phosphate pyrophosphorylase [Cordyceps militaris] ATY65286.1	1	ACA1_061790				
	Thiamine-phosphate pyrophosphorylase [Marchantia polymorpha subsp. ruderalis] BFI36074.1	2	ACA1_061800				
			ACA1_061790				
Alkaline phosphatase	Alkaline phosphatase [Ipomoea batatas] GMD03768.1	2	ACA1_182290	alkaline phosphatase D domain containing protein	Alkaline phosphatase	XP_004345909.1	Yes
			ACA1_097100	alkaline phosphatase D, putative	Alkaline phosphatase	XP_004335040.1	No
	Alkaline phosphatase [Talaromyces islandicus] CRG90206.1	2	ACA1_194910	alkaline phosphatase family subfamily protein	Alkaline phosphatase	XP_004367444.1	Yes
			ACA1_373910	alkaline phosphatase family subfamily protein	Alkaline phosphatase	XP_004334337.1	No
Thiamine pyrophosphokinase	Thiamine pyrophosphokinase [Cocos nucifera] KAG1338861.1	0					
	Thiamine_pyrophosphokinase [Candida] auris] XP_028893050.2	2	ACA1_225950	thiamine pyrophosphokinase	Thiamine pyrophosphokinase	XP_004346474.1	Yes
			ACA1_050760	thiamine pyrophosphokinase	Thiamine pyrophosphokinase	XP_004334518.1	Yes
		2	ACA1_225950				

	Thiamine pyrophosphokinase [Halanaerobium praevalens DSM 2228] ADO76910.1		ACA1_050760				
Nucleoside triphosphate	TPA: nucleoside triphosphate pyrophosphohydrolase HAM1 [Saccharomyces cerevisiae S288C]	1	ACA1_204490	Ham1 family	Hypothetical protein/inosine triphosphate pyrophosphatase	XP_004338375.1	Yes
	MAG: NTPase [Anaerolineae bacterium] MCS7287447.1	1	ACA1_129200	Nucleoside-triphosphatase	Nucleoside-triphosphatase	XP_004336830.1	Yes
	Nucleoside triphosphate pyrophosphohydrolase ham1 [Aspergillus luchuensis] XP_041547643.1	1	ACA1_204490				

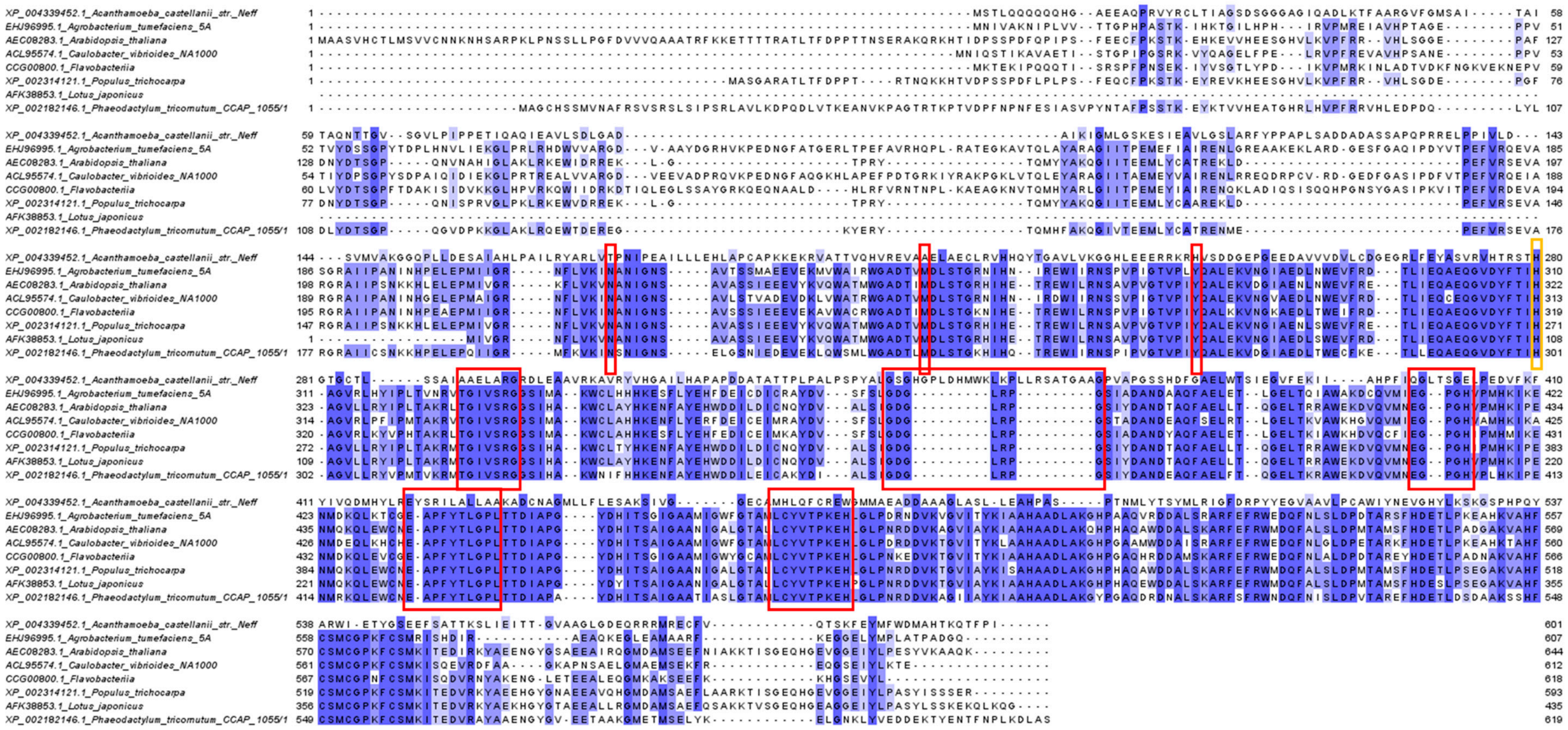

Figure S1: Sequence alignment of the putative *Acanthamoeba* hydroxymethylpyrimidine kinase gene ACA1_061800 with those found in other organisms. Active site locations are located within the coloured boxes, those coloured red were absent from the gene ACA1_061800, those coloured orange were present.

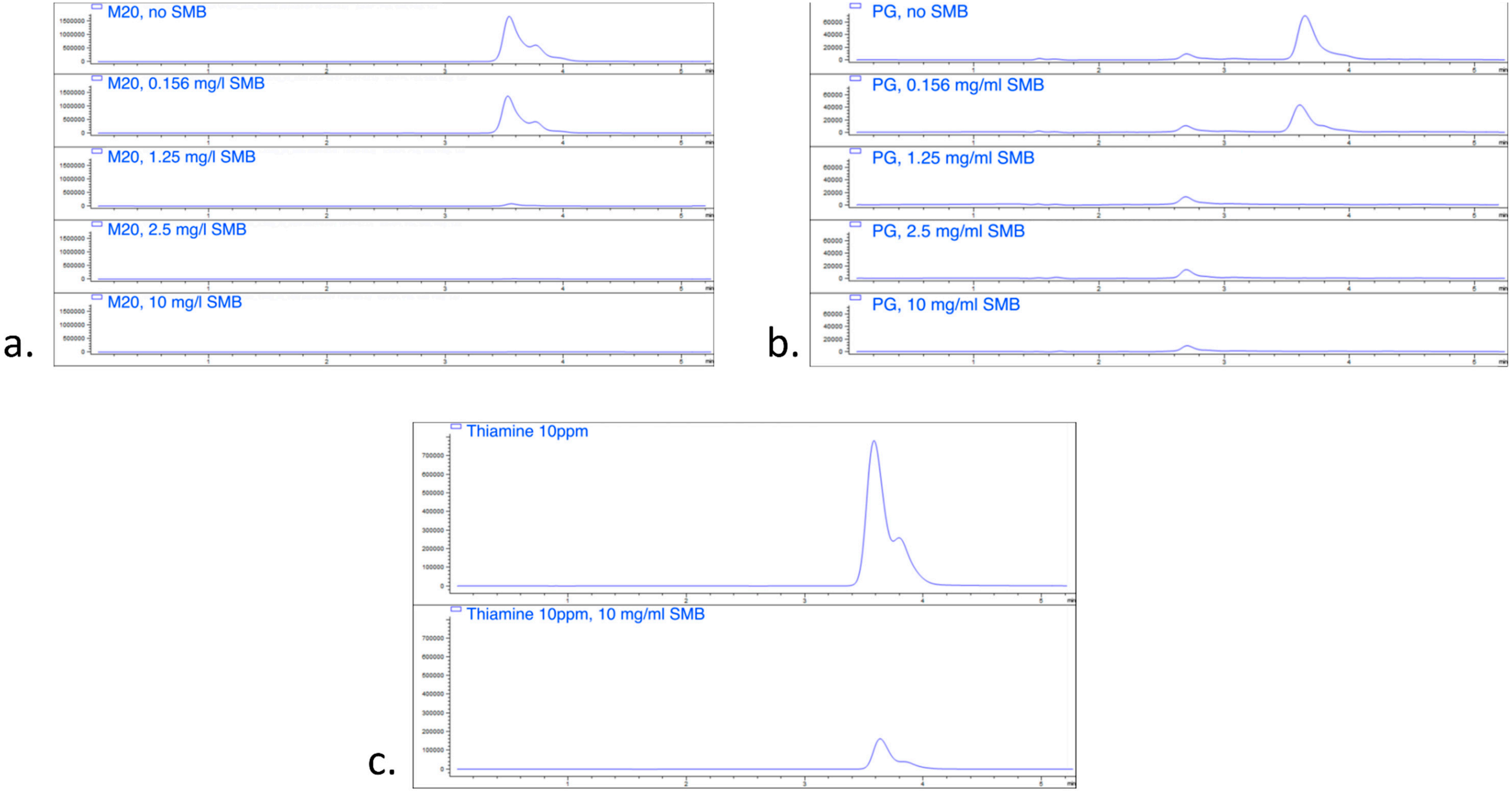

Figure S2. LC-MS was used to detect thiamine levels in a. thiamine rich M20 media (1.25 mg/ml) and b. PG media after 24 hours incubation with or without sodium metabisulfite at concentrations of 0 mg/ml, 0.156 mg/ml, 1.25 mg/ml, 2.5 mg/ml, and 10 mg/ml and c. a thiamine standard (10 ppm) when incubated with or without 10 mg/ml of sodium metabisulfite. Peak height is indicative of available thiamine in the media. Thiamine is depleted in M20 media in a dose dependent manner when incubated with sodium metabisulfite.
