# Supplementary figures and images for: Sodium Metabisulfite Inhibits Acanthamoeba Trophozoite Growth through Thiamine Depletion
Source: Pathogens. 2024 May 21;13(6):431. doi: 10.3390/pathogens13060431 (PMC11206890; doi:10.3390/pathogens13060431)

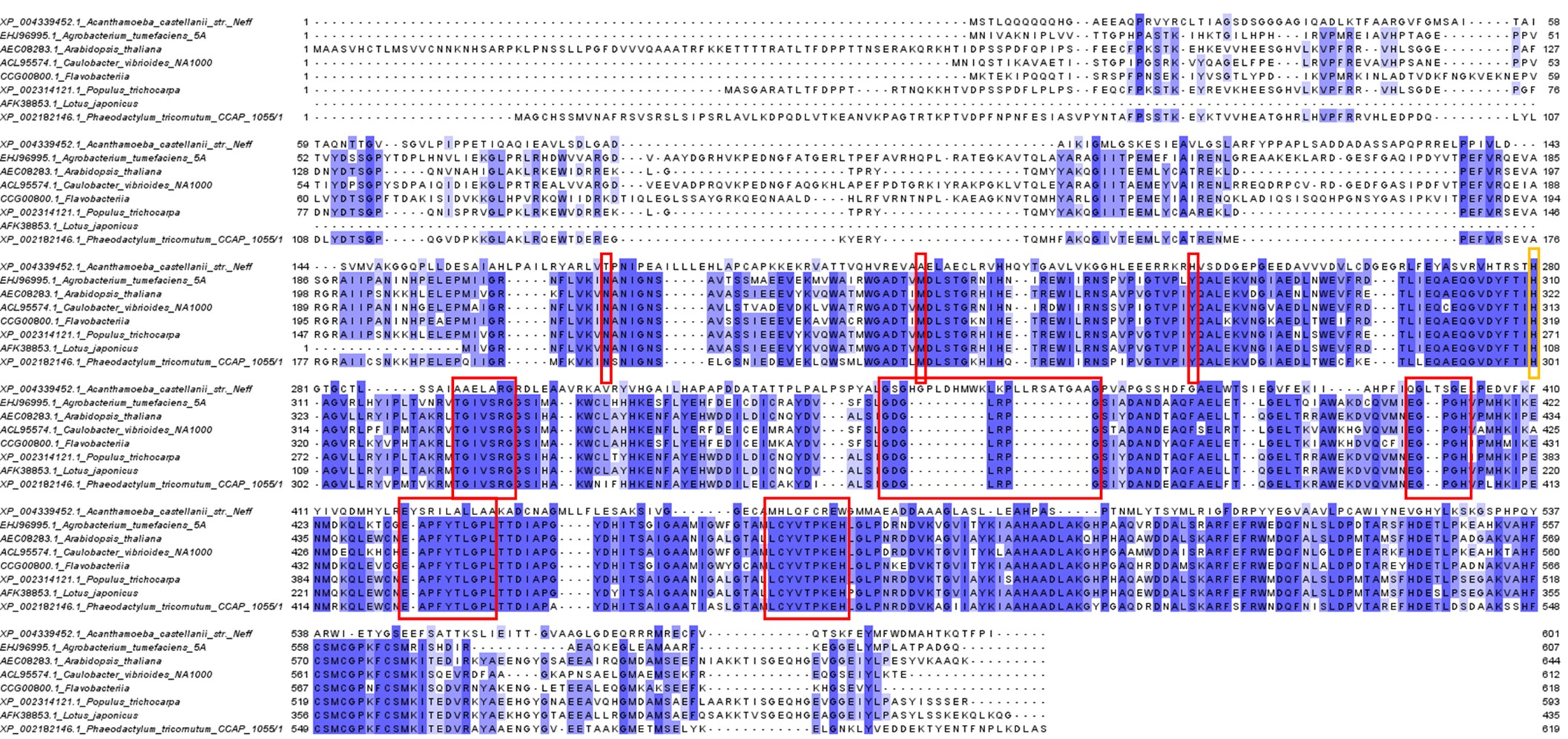

Supplement: Supplementary file 1 [file pathogens-13-00431-s001.zip › Figure S1.jpg]

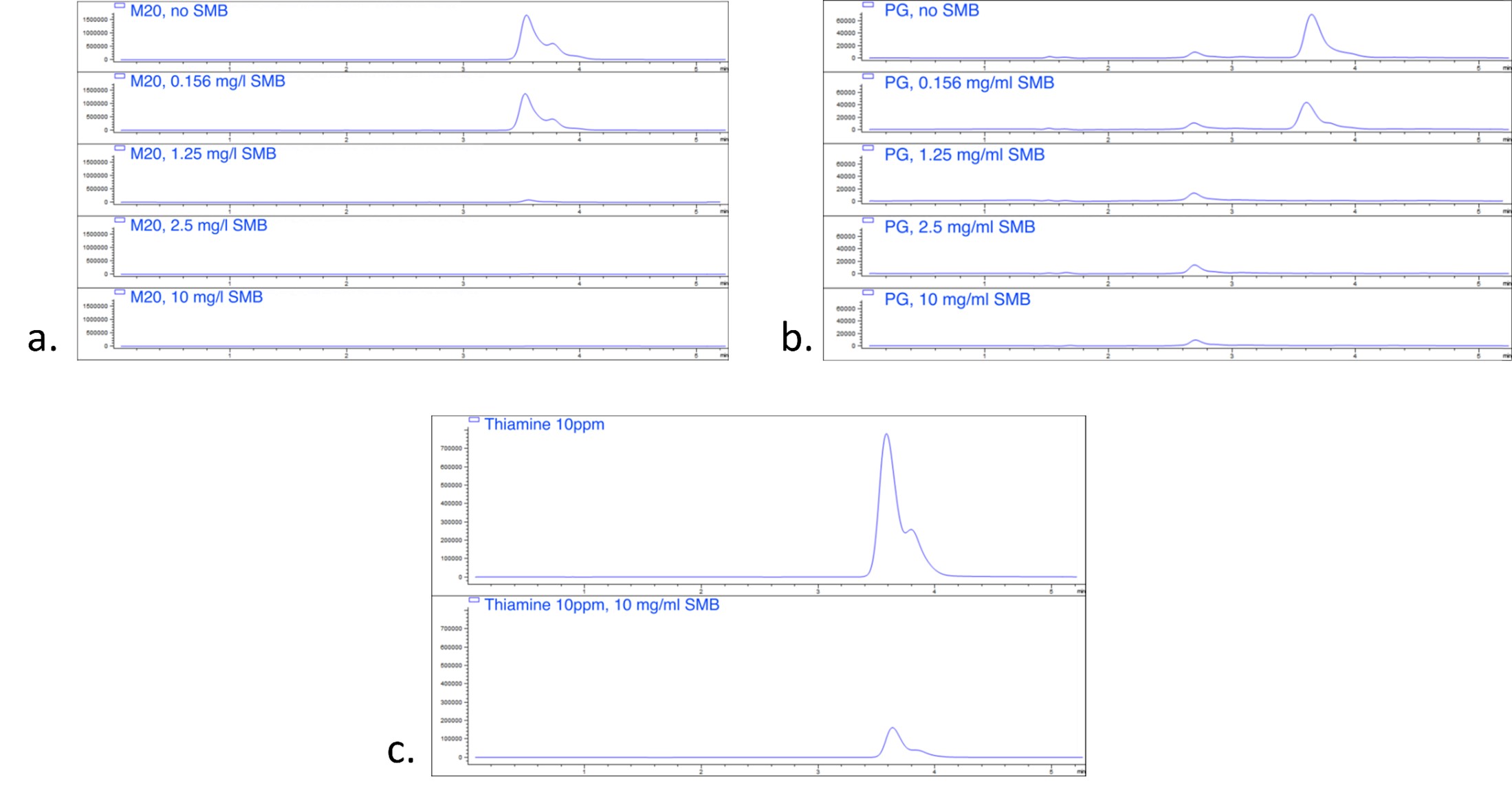

Supplement: Supplementary file 1 [file pathogens-13-00431-s001.zip › Figure S2.jpg]
